# Prevalence of under-nutrition and its associated factors among 6–23 months old children of employed and unemployed mothers in town kebeles of Dera district, northwest Ethiopia: a comparative cross-sectional study

**DOI:** 10.1186/s40795-023-00713-0

**Published:** 2023-03-28

**Authors:** Habtamu Zelalem, Yeshalem Mulugeta Demilew, Samuel Dagne, Anteneh Mengist Dessie

**Affiliations:** 1 Department of Nutrition and Dietetics, Felege Hiwot Comperhensive Specialized Hospital, Bahir Dar, Ethiopia; 2 Department of Nutrition and Dietetics, School of Public Health, College of Medicine and Health Science, Bahir Dar University, Bahir Dar, Ethiopia; 3 Department of Public Health, College of Health Sciences, Debre Tabor University, Debre Tabor, Ethiopia

**Keywords:** Under-nutrition, 6–23 months Old Children, Maternal employment, Dera district, Northwest Ethiopia

## Abstract

**Background:**

Child under-nutrition remains a widespread problem around the globe. Improving child nutrition and empowering women are two important and closely connected development goals. These two interconnected goals will affect one another through different mechanisms, and the net effect may not necessarily be positive. Yet, the impact of maternal employment, one method of empowering mothers, on children’s nutritional is not well studied in Ethiopia. Hence, this study is to compare the prevalence of under-nutrition and its associated factors among 6–23 months old children of employed and unemployed mothers in town kebeles of Dera district, Northwest Ethiopia, 2022.

**Methods:**

A community-based comparative cross-sectional study design was conducted among 356 employed and 356 unemployed mothers having 6–23 months old children. A systematic random sampling technique was used to select study participants. Epi-data version 3.1 and SPSS version 25.0 statistical software were used for data entry and analysis, respectively. Both bi-variable and multivariable binary logistic regression was done to assess the association between independent and dependent variables. A p-value of less than 0.05 in a multivariable binary logistic regression was declared as the level of statistical significance.

**Result:**

The prevalence of under-nutrition was 69.8% (95% CI: 65.0, 74.7) among children of unemployed mothers, compared to 27.4% (95% CI: 22.7, 32.2) among children of employed mothers. Under-nutrition among children of unemployed mothers was significantly associated with being a male child, age increase by one month, household food insecurity, lack of ANC follow-up, and not exclusively breastfed. Whereas, among children of employed mothers, being a male child, age increase by one month, being sick during the last two weeks prior to data collection, not immunized to their age, and low meal frequency were significantly associated with their under-nutrition.

**Conclusion:**

The prevalence of under-nutrition among children of unemployed women is significantly higher than those children of employed women which consolidate the evidence that women’s employment status have a positive association with child nutrition. Different factors were also identified as significant predictors of child under-nutrition among these two groups (employed and unemployed women). Thus, multi-sectoral intervention approach together with agriculture and education offices should be strengthened.

## Background

Adequate nutrition in early childhood is essential to ensure healthy growth, proper organ formation and function, a strong immune system, and neurological and cognitive development. Particularly, the first 1000 days of a child’s life, from conception to their second birthday, are the most crucial in their development and the window of opportunity for guaranteeing their survival, flourishing, and full potential [1]. Everyone wants their child to be healthy and feel good about themselves, both mentally and physically. Thus, considering the importance of nutrition in the child’s development, especially in the first two years of life, will have an effect on their health, both now and in the future [2].

However, child under-nutrition remains a widespread problem and a major development challenge in many low- and middle-income countries (LMICs). It is the underlying cause of death in an estimated 45% of all child deaths [3, 4]. Every hour and minute of every day, 300 and 5 children die because of malnutrition, respectively [5]. The magnitude of stunting, wasting, and underweight among children in LMICs was 29.1%, 6.3%, and 13.7% respectively [6]. The recent mini Ethiopian Demographic Health Survey (mEDHS) report also indicates 37%, 7% and 21% of children under five years old were stunted, wasted and underweight, respectively [7].

Ethiopia has made substantial progress in addressing malnutrition over the last decade. The National Nutrition Program (NNP) has gradually expanded in scope since its 2008 launch [8]. The Sequota declaration implementation plan aimed at ending under-nutrition by 2030 has fostered work across sectors. Now, the Ethiopian government launched the National Food and Nutrition Policy (NFNP), which promotes a coordinated and comprehensive approach to food and nutrition security and stresses the importance of access to nutritious foods. There is also a National Food and Nutrition Strategy that was driven from the food and nutrition policy of Ethiopia, taking into consideration that the overarching policy directions were unpacked into strategic objectives, strategic directions, and strategic initiatives.The NFNP is expected to play a major role in addressing Ethiopia’s nutrition challenges. Yet many children remain under-nourished and it is still a big problem of the health system in Ethiopia.

The causes of malnutrition are classified as immediate, underlying and basic [9]. Women’s status is considered as one of the basic cause for malnutrition. It is widely accepted that the work status of the mother plays an important role in determining nutrition status of her child [10, 11]. The relationship between maternal work status and nutritional status of children are complex issue surrounded with controversies. Two separate mechanisms for this effect have been postulated [12]: first, the mother’s higher income as a result of her employment, and second, the time she spends away from child care when she goes to work. While the first has a direct and positive effect on the nutritional status of her child, the second has a negative effect [13].

The Ethiopian government policy on women aims at creating an environment that promotes women to be employed. As a result, the proportion of women in the workforce is gradually increasing. It would therefore be fascinating to find out whether working mothers’ time limitations or their income earned by working, particularly in town kebeles, have a greater impact on children’s nutritional status. However, the prevalence of undernutrition in children aged 6 to 23 months and the contributing factors for this problem have not been thoroughly investigated, particularly the relationship between mothers’ employment status and children’s nutritional status. With this understanding, in this study, child under-nutrition was examined in relation to maternal employment status in Dera district, Northwest Ethiopia.

## Methods

### Study setting and period

This study was conducted from 23 May to 23 June 2022 at town kebeles of Dera district which is one of fifteen districts in South Gondar zone, Amhara region, Ethiopia. It is located at 594 Km Northwest of Addis Ababa, the capital city of Ethiopia and 42 Km from Bahir Dar, the central city of the Amhara region. Dera district is bordered on the south by the Abay River which separates it from the West Gojam Zone, on the west by Lake Tana, on the north by Fogera district, on the northeast by East Este, and on the east by West Este. The district comprises three town kebeles with an estimated total population of 40,750 people. Children under 2 years of age are expected to constitute 2,119 (5.2%) of the total population. The farming system of the area is mixed crop-livestock production. Crop production covers a wide range of crops which include both cash and food crops such as rice, maize, millet, teff, barley, and potato [14].

### Study design

A community-based comparative cross-sectional study design was used in the research.

### Eligibility criteria

All children aged 6 to 23 months whose mother had been residents of the study areas for six and more months were included in the study. Those children who had physical deformities that hinders all methods of length measurements were also excluded.

### Sample size determination

The minimum required sample size was determined by using Epi Info statistical software version 7 using two population proportion formulas. The following assumptions were taken into consideration: confidence level = 95%, power = 80%, ratio = 1:1, the proportion of under-weight among 6 to 23 months old children of unemployed mothers of 13.8% and that of children of employed mothers 7.2% taken from the study done at Addis Ababa [15]. Considering a 5% possible non-response rate, 356 unemployed and 356 employed mothers were included.

### Sampling procedure

The list of all children aged 6 to 23 months with their corresponding house number in the three town kebeles of Dera district was obtained from Health Extension Workers. Sampling frame was prepared separately for employed and unemployed mothers of having those children after determining their employment status by going to the house of all listed children. Then the calculated sample size was allocated to each kebele proportional to their size of the population. Finally, systematic random sampling technique was applied to select the study participants after determining the interval (K = 2 & 3 for Employed and Unemployed, respectively) by dividing the total eligible children in the kebele to the allocated sample proportion. Whenever there was more than one child aged 6 to 23 months in a household, the youngest child was considered.

### Operational/term definition

#### Employment status

A mother is considered “employed” if she reports having had a paid job with a specified salary in a government, NGO, or private sectors for the last six or more months. Otherwise, a woman was considered “unemployed.”

#### Under-nutrition

Refers to a child who has stunting, wasting, and/or underweight. Children whose WLZ is less than − 2SD of the WHO standard were classified as wasted, their weight for the age of Z < − 2SD were labeled as underweight, and their length for the age of Z < − 2SD were considered stunted [16].

#### Source of drinking water

Improved water source is piped water, piped dwelling, piped into compound, piped outside the compound, tube well or borehole, dug well, protected well and protected spring. While non- improved water source is unprotected well, unprotected spring, rainwater, and tanker truck, surface water (river, dam, and lake/pond) [17].

#### Food security

Food security status was assessed using 18 previously validated questions (9 occurrences and 9 frequencies of occurrence questions). Since the frequency-of occurrence questions have only three response options representing a range of frequencies (1 = rarely, 2 = sometimes, 3 = often) it was coded as 0 for all cases where the answer to the corresponding occurrence question was “no”. Then those with Household Food Insecurity Access (HFIA) category one were categorized as food secure, and those in HFIA categories two through four were considered food insecure [18].

#### Latrine facility

Improved, not shared is flush or pour flush toilet, flush to piped sewer, flush to septic tank, flush to pit latrine, ventilated improved pit latrine (VIP), and pit latrine with slab. While non- improved toilet facility is flush to somewhere else, pit latrine without slab, open pit latrine, composing toilet, bucket toilet, hanging toilet/hanging latrine, no facility/bush/field [17].

#### Minimum dietary diversity

Children 6–23 months of age who consume 5 or more of the 8 food groups (grains, roots and tubers; legumes and nuts; dairy products (milk, yogurt); flesh food (meat, fish, poultry and liver/organ meats); eggs; vitamin A rich fruits and vegetables; other fruits and vegetables; and breast milk) with 24 h dietary recall were considered as having minimum dietary diversity [19].

#### Minimum meal frequency

Children of age 6–8 months fed a minimum of three meals per day and children aged 9 months and above fed four times per day within a 24-hourdietary recall period were considered as having minimum meal frequency [19].

#### Minimum acceptable diet

Is a composite indicator of minimum dietary diversity and minimum meal frequency, which indicates the proportion of children 6–23 months of age who received a minimum diversified diet and minimum meal frequency (apart from breast milk) [19].

### Data collection tool and technique

Data were collected using an Amharic version interviewer-administered pre-tested structured questionnaire, which was prepared after reviewing various available literatures [15, 20–24]. The instrument comprises socio-demographic variables, variables used to assess wealth of the household, food security, child characteristics, maternal service utilization and working characteristics, child feeding practice, and anthropometry measurement and related information. Data for the IDDS indicator was collected by asking the respondent a series of yes-or-no questions. These questions were asked to the person who is responsible for food preparation or feeding the child, usually the mother. A 24-hour recall method was used to reduce the cjance of n less accurate information due to imperfect recall. Body lengths and weights of the children were measured immediately following the interviews of the mothers to the nearest 0.1 cm and 0.1 kg using a horizontal wooden length board and salter scale, respectively, with the child wearing light clothing and no shoes. Mid Upper Arm Circumference (MUAC) was also measured on left arm, half way between the olecranon and acromion process using non stretchable tap to the nearest 1 mm. These measurements were taken two times and the average was recorded. The ages of children were ascertained by comparing with child’s birth.

### Data processing and analysis

Data were entered into Epi-data version 3.1 and exported to SPSS version 25.0 for further analysis. Anthropometric measurements were converted into Z-score of length for age, weight for length and weight for age by using WHO Anthro software for analysis. The wealth index of the household was determined using principal component analysis (PCA) by considering latrine, water source, housing condition, house ownership, cooking methods, household assets, and bank account. The responses of all variables were recoded as 0 and 1, with the higher value receiving a code of 1 and the smaller value receiving a code of 0. Factor scores were produced using variables having a commonality value of greater than 0.5 in PCA. Finally, three quintiles that divide the household in to three groups (poor, medium, and rich) were constructed using the first principal component. Descriptive statistics such as frequencies, proportions, and cross-tabulation was used to present the data. Bi-variable binary logistic regression was done to assess the association between independent and dependent variables. Variables that showed association (p-value ≤ 0.25) in the bi-variable analysis were included in the multivariable binary logistic regression model. These variables with p value of less than 0.05 at 95% confidence level in the multivariable binary logistic regression were considered as significant. A Hosmer and Lemeshow test was used to evaluate the model fitness and the results showed that all three models were well-fitted with a p value of > 0.05.

## Results

### Socio-demographic characteristics of respondents

Seven hundred twelve mothers having children aged 6–23 months were planned to be interviewed in this study. Out of these, 687 gave a complete response in the actual data collection making a response rate of 96.5% of which 339 (49.3%) were employed and 348 (50.7%) were unemployed mothers. Pertaining to the educational status of mother, 315 (92.9%) of employed and 15 (4.3%) of unemployed mothers were attended college and above, whereas none of employed and 66 (19.0%) of unemployed mothers were unable to read and write. The median age of employed mothers were 32 years with Inter Quantile Range (IQR) of 6 (36 − 30) and unemployed mothers were 34 years with IQR of 8 (38 − 30). The family of 273 (80.5%) and 156 (44.8%) employed and unemployed women were food-secured, respectively (Table 1).

**Table 1:** Socio-demographic characteristics of respondents in town kebeles of Dera district, northwest Ethiopia, 2022

Variables	Employed N (%)	Unemployed N (%)	*P-value*
Religion			
Orthodox	249 (73.5)	246 (70.7)	0.001
Muslim	78 (23.0)	102 (29.3)	
Protestant	12 (3.5)	0 (0.0)	
Marital status of mothers			
Married	303 (89.4)	324 (93.1)	0.037
Divorced	18 (5.3)	18 (5.2)	
Widowed	18 (5.3)	6 (1.7)	
Educational status of mothers			
Can’t read and write	0 (0.0)	66 (19.0)	< 0.001
Read and write only	0 (0.0)	108 (31.0)	
Primary school	3 (0.9)	93 (26.7)	
Secondary school _ Grade 9–12	21 (6.2)	66 (19.0)	
Higher level _ Diploma & above	315 (92.9)	15 (4.3)	
Educational status of fathers			
Can’t read and write	0 (0.0)	24 (7.0)	< 0.001
Read and write only	7 (2.2)	108 (31.6)	
Primary school	59 (18.4)	84 (24.6)	
Secondary school _ Grade 9–12	80 (24.9)	72 (24.1)	
Higher level _ Diploma & above	175 (54.5)	54 (15.8)	
Occupation of fathers			
Government employee	169 (52.6)	51 (14.9)	< 0.001
Private employee	29 (9.0)	24 (7.0)	
Private business	120 (37.4)	159 (46.5)	
Daily laborer	3 (0.9)	30 (8.8)	
Farmer	0 (0.0)	78 (22.8)	
Wealth of the household			
Poor	15 (4.4)	216 (62.1)	< 0.001
Medium	135 (39.8)	84 (24.1)	
Rich	189 (55.8)	48 (13.8)	
Household Food Insecurity Access Scale			
Food Secure	273 (80.5)	156 (44.8)	< 0.001
Food Insecure	66 (19.5)	192 (55.2)	
Head of the Household			
Father	3 (0.9)	66 (19.0)	< 0.001
Mother	60 (17.7)	33 (9.5)	
Both	276 (81.4)	249 (71.6)	
Number of family members			
< 5	177 (52.2)	114 (32.8)	0.728
5 and above	162 (47.8)	234 (67.2)	
Number of Under-5 children			
One	174 (51.3)	174 (50.0)	< 0.001
Two and above	165 (48.7)	174 (50.0)	

### Childs’ characteristics

One hundred sixty five (48.7%) employed and 135 (38.8%) unemployed mothers had children within the age range of 18–23 months. One hundred sixty two (47.8%) and 168 (48.3%) children of employed and unemployed mother were females. Moreover, 129 (38.1%) and 138 (39.7%) children of employed and unemployed were got sick in the past two weeks prior to the data collection period (Table 2).

**Table 2:** Childs’ characteristics by employment status of mother in town kebeles of Dera district, northwest Ethiopia, 2022

Variables	Employed N (%)	Unemployed N (%)	*P-value*
Age of children (in month)			
6–11	75 (22.1)	90 (25.9)	0.033
12–17	99 (29.2)	123 (35.3)	
18–23	165 (48.7)	135 (38.8)	
Sex of children			
Male	177 (52.2)	180 (51.7)	0.898
Female	162 (47.8)	168 (48.3)	
Birth order			
First	57 (16.8)	15 (4.3)	0.001
Second	156 (46.0)	159 (45.7)	
Third and above	126 (37.2)	174 (50.0)	
Birth interval (inter-birth)			
Not applicable	57 (16.8)	15 (4.3)	< 0.001
< 33 months	171 (50.4)	282 (81.0)	
33 and above months	111 (32.7)	51 (14.7)	
Child got sick in the last 2 weeks prior to data collection			
Yes	129 (38.1)	138 (39.7)	0.667
No	210 (61.9)	210 (60.3)	
Immunization status			
Vaccinated to their age	281 (82.9)	139 (39.9)	< 0.001
Not vaccinated to their age	58 (17.1)	209 (60.1)	

### Maternal service utilization and working characteristics

As to maternal health service utilization characteristics of study participants, 336 (99.1%) and 174 (50%) employed and unemployed mothers attended antenatal care for their last pregnancy, respectively. Whereas, only 258 (76.1%) and 72 (20.7%) employed and unemployed mothers attended postnatal care for their last pregnancy. The majority 336 (99.1%) of employed and 276 (79.3%) of unemployed mothers gave birth to the indexed child at health institutions. Almost all 336 (99.1%) employed mothers were working for 5–6 days/week for 8 h/day, and 306 (90.3%) of the mothers reported that their workplace is not convenient (have no feeding room and not give time to feed). Two hundred nineteen (64.6%) employed mothers leave the child with an alternate caregiver while they go to work.

### Child feeding practice

About 303 (89.4%) of employed and 207 (59.5%) of unemployed mothers initiate breast-feeding during the first hour after delivery. Two hundred ninety seven (87.6%) employed and 297 (85.3%) unemployed mothers were breastfed their children till the time of the survey. Regarding the use of bottle-feeding which has nipple, 114 (33.6%) were employed and 87 (25.0%) were unemployed. When mothers asked about child feeding practice, from eight food groups which are listed in WHO indicators for assessing infant and young child feeding practice guideline, 267 (78.8%) employed and 56 (16.1%) unemployed mothers fed five or more food groups for their children. Coming to the feeding frequency, only 264 (77.9%) employed and 90 (25.9%) unemployed mothers feed their children with adequate frequency for their age.

### Nutritional status of children

The result of Anthropometry demonstrates that the prevalence of wasting, stunting, and underweight among 6–23 month old children of both employed and unemployed mother was 10.9%, 38.9%, and 29.3%, respectively. Children of employed mothers had under nutrition rate of 27.4% (95% CI: 22.7, 32.2), compared to those of unemployed mothers, which was 69.8% (95% CI: 65.0, 74.7) (Table 3).

**Table 3:** Nutritional status of 6–23 months old children of employed and unemployed mother in town kebeles of Dera district, northwest Ethiopia, 2022

Variables	Employed N (%)	Unemployed N (%)	Total N (%)
***Wasting***			
Over (> 2 SD)	36 (10.6)	51 (14.7)	87 (12.7)
Normal (+ 2 to -2SD)	291 (85.8)	234 (67.2)	525 (76.4)
Moderate wasting (<-2 SD to -3SD)	9 (2.7)	33 (9.5)	75 (6.1)
Sever wasting (<-3 SD)	3 (0.9)	30 (8.6)	33 (4.8)
***Underweight***			
Over (> 2 SD)	6 (1.8)	9 (2.6)	15 (2.2)
Normal (+ 2 to -2SD)	291 (85.8)	180 (51.7)	471 (68.6)
Moderate Underweight (<-2 SD to -3SD)	30 (8.8)	93 (26.7)	123 (17.9)
Sever underweight (<-3 SD)	12 (3.5)	66 (19.0)	78 (11.4)
***Stunting***			
Over (> 2 SD)	18 (5.3)	15 (4.3)	33 (4.8)
Normal (+ 2 to -2SD)	234 (69.0)	153 (44.0)	387 (56.3)
Moderate Stunting (<-2 SD to -3SD)	36 (10.6)	48 (13.8))	84 (12.2)
Sever stunting (<-3 SD)	51 (15.0)	132 (37.9)	183 (26.6)
***Under nutrition (stunted, wasted, and/or underweight)***			
Yes	93 (27.4)	243 (69.8)	336 (48.9)
No	246 (72.6)	105 (30.2)	351 (51.1)

### Factors associated with nutritional status of children

Multivariable binary logistic regression of the overall model with the following variables: maternal employment status, child age, child sex, ANC follow up, birth place, child immunization status, got sick in the last two weeks prior data collection, breast feeding initiation time, bottle feeding, exclusive breast feeding, number of under-five children in the family, household wealth index, food security, food diversity, and feeding frequency were fitted. As the result of this regression indicates, maternal employment (AOR = 2.96, 95% CI = (1.64, 5.35)), being male (AOR = 4.34, 95% CI = (2.79, 6.75)), increase in child age by one month (AOR = 0.856 95% CI = (0.81, 0.90)), haven’t ANC follow up (AOR = 2.22, 95% CI = (1.16, 4.23)), child got sick in the last two weeks prior data collection (AOR = 2.46, 95% CI = (1.54, 3.94)), being food insecure (AOR = 6.86, 95% CI = (3.97, 11.87)), inappropriate frequency of feeding (AOR = 1.71, 95% CI = (1.02, 2.87)), and less diversified food intake (AOR = 3.06, 95% CI = (1.72, 5.42)) were found to be significant predictors of child under-nutrition. This significant difference in child under nutrition among employed and unemployed women after adjusting with the above-listed variables enables us to do a comparative study and therefore run two different regression models for each group in order to separately identify the associated factors.

Accordingly, for children of employed mothers, household wealth index, number of under-five children, family size, age of the child, sex of the child, got sick in the last 2 weeks, immunization status, PNC follow-up for the last birth, bottle feeding, and meal frequency were found to be significant variables at alpha 0.25 in the bi-variable binary logistic regression analysis. These varaibles were selected as a candidate variables for multivariable binary logistic regression analysis. The multivariable regression result indicated that being male (AOR = 6.02, 95% CI = (2.99, 12.11)), child got sick in the last two weeks prior data collection (AOR = 6.17, 95% CI = (3.09, 12.30)), not immunized to their age (AOR = 4.66, 95% CI = (1.87, 11.59)), and inappropriate feeding frequency (AOR = 4.60, 95% CI = (2.09, 10.16)) were found to have significant positive association with under-nutrition among 6–23 month old children of employed mothers. Whereas, an increase in child age by one month (AOR = 0.86, 95% CI = (0.80, 0. 0.92)) decrease the odds of under-nutrition (Table 4).

**Table 4:** Factors associated with under nutrition of 6–23 month old children of employed mothers in town kebeles of Dera district, northwest Ethiopia, 2022

Variables	Under-nutrition		COR (95%CI)	AOR (95%CI)
	Yes	No		
*Household wealth*				
Poor	9	6	5.77 (1.94, 17.18)	3.38 (0.52, 21.83)
Medium	45	90	1.92 (1.16, 3.17)	1.71 (0.85, 3.41)
Rich	39	150	1	1
*Number of Under-5 children*				
One	42	132	1	1
Two and above	51	114	1.41 (0.87, 2.27)	1.92 (0.92, 3.99)
*Family size*				
< 5	57	120	1	1
5 and above	36	126	0.60 (0.370, 0.98)	0.92 (0.48, 1.79)
Child sex				
Male	72	105	4.60 (2.66, 7.96)	6.02 (2.99, 12.11)*
Female	21	141	1	1
*Child age*				
			0.91 (0.87, 0.96)	0.86 (0.80, 0.92)*
*Child got sick in the last 2 weeks*				
Yes	57	72	3.83 (2.32, 6.31)	6.17 (3.09, 12.30)*
No	36	174	1	1
*Immunization status*				
Vaccinated to their age	57	224	1	1
Not vaccinated to their age	36	22	6.43 (3.51, 11.77)	4.66 (1.87, 11.59)*
*PNC follow-up*				
Yes	63	195	1	1
No	30	51	1.82 (1.07, 3.10)	0.47 (0.18, 1.21)
*Bottle Feeding*				
Yes	30	57	1	1
No	63	189	0.63 (0.37, 1.07)	1.20 (0.52, 2.77)
*Feeding frequency*				
Appropriate for age	54	210	1	1
Not appropriate for age	39	36	4.21 (2.45, 7.25)	4.60 2.09, 10.16)*

Bold and * = significant at P < 0.05; COR = Crude Odds Ratio; AOR = Adjusted Odds Ratio; CI = Confidence Interval

Coming to the children of unemployed mothers, household wealth index, food security, ANC follow-up and post-natal service utilization for the indexed child, age of the child, sex of the child, dietary diversity, meal frequency, got sick in the last 2 weeks, breast feeding initiation time and exclusive breast feeding status were selected as a candidate variable for multivariable binary logistic regression analysis models. Accordingly, being male (AOR = 3.40, 95% CI = (1.91, 6.04)), food insecure (AOR = 5.80, 95% CI = (2.95, 11.43)), not having ANC follow-up during pregnancy (AOR = 3.71, 95% CI = (1.90, 7.25)), and not exclusively breast feed the child till six months (AOR = 2.29, 95% CI = (1.19, 4.40)) were positively associated with under nutrition of 6–23 month old children of unemployed mothers, while an increase in age of the child by one month (AOR = 0.89, 95% CI = (0.84, 0.95)) decrease the odds of under nutrition among these children (Table 5).

**Table 5:** Factors associated with under nutrition of 6–23 month old children of unemployed mothers in town kebeles of Dera district, northwest Ethiopia, 2022

Variables	Under-nutrition		COR (95%CI)	AOR (95%CI)
	Yes	No		
*Household wealth*				
Poor	156	60	2.02 (1.06, 3.85)	0.42 (0.11, 1.55)
Medium	60	24	1.94 (0.93, 4.08)	0.99 (0.29, 3.31)
Rich	27	21	1	1
*Household Food Insecurity Access Scale*				
Food secure	81	75	1	1
Food insecure	162	30	5.00 (3.03, 8.25)	5.80 (2.95, 11.43)*
*Dietary diversity*				
≥ 5 food groups	32	24	1	1
< 5 food groups	211	81	1.95 (1.09, 3.52)	1.47 (0.46, 4.62)
*Child sex*				
Male	141	39	2.34 (1.46, 3.75)	3.40 (1.91, 6.04)*
Female	102	66	1	1
*Child age*				
Managed as continuous variable			0.95 (0.91, 1.01)	0.89 (0.84, 0.95)*
*Child got sick in the last 2 weeks*				
Yes	111	27	2.43 (1.47, 4.03)	1.20 (0.64, 2.26)
No	132	78	1	1
*ANC follow-up*				
Yes	96	78	1	1
No	147	27	4.42 (2.66, 7.35)	3.71 (1.90, 7.25)*
*PNC follow-up*				
Yes	45	27	1	1
No	198	78	1.52 (0.88, 2.63)	0.46 (0.22, 1.06)
*Breast feeding initiation*				
Within 1 h of delivery	135	72	1	1
After 1 h of delivery	108	33	1.75 (1.08, 2.83)	0.98 (0.54, 1.79)
*Six months exclusive breast feeding*				
Yes	93	54	1	1
No	150	51	1.71 (1.08, 2.71)	2.29 (1.19, 4.40)*

Bold and * = significant at P < 0.05; COR = Crude Odds Ratio; AHR = Adjusted Odds Ratio; CI = Confidence Interval

## Discussion

The present study was designed to compare the under nutrition of children aged 6–23 months and its associated factors among employed & unemployed mothers in Dera district, Northwest Ethiopia. In this study, the prevalence of stunting, underweight, and wasting among children of employed women was 25.7%, 12.4%, and 3.5%, respectively, whereas the prevalence among children of unemployed women was 51.7%, 45.7%, and 18.1%. The overall prevalence of under nutrition was also significantly higher among children of unemployed mother 69.8% (95% CI: 65.0, 74.7) compared to 27.4% (95% CI: 22.7, 32.2) among children of employed mother. This indicated that under nutrition is more common among children of unemployed women, supporting one of the widely accepted premises that the positive impact of a higher income due to mother’s employment on the nutritional status of her child outweighs the adverse impact of the time she spends away from childcare.

The finding from children of employed mothers is lower than 2019 mEDHS report that is stunting (37%), underweight (21%), and wasting (7%). On the other hand, prevalence of these under-nutrition measures among children of unemployed women is higher than this 2019 mEDHS report [7]. This is could be due to EDHS has been consider both employed and unemployed mothers together. It has a big implication that maternal employment status has a significant effect on child nutrition and children of employed women are less likely to develop under nutrition as compared to children of unemployed women.

The prevalence of stunting (58.1%) and wasting (17.0%) among children aged 6–24 months in Dabat health and demographic surveillance system site [25] is in line with our finding from children of unemployed women, but higher than the finding from children of employed women. The possible reason for the above variation could be the population difference and a time gap between the studies as there is intensive nutritional intervention by the government as well as the non-governmental organization recently, which possibly contributed to the reduction of under nutrition especially among children of employed mothers. Yet, the prevalence of stunting, underweight, and wasting among both children of employed and unemployed women is totally higher than the study conducted in Addis Ababa, which reports 6.8%, 7.2%, and 5.8% of stunting, under-weight, and wasting in children of employed mothers and 12.9%, 13.8%, and 8.1% in the children of unemployed mothers, respectively [15]. This variation could be justified by the difference in study area, where Addis Ababa, Ethiopia’s capital and largest city, has a population that lives in a quite different environment and lifestyle from the urban settings of particular districts and also there is more focus and attention in nutrition intervention activities in Addis ababa from the government and NGO`s.

Although there are some similar factors (age and sex of the child) which were significantly associated with child under nutrition of both employed and unemployed mothers, some unique factors are also there. While child under-nutrition among unemployed mothers was affected by food security status, ANC follow-up during pregnancy, and six month exclusive breast feeding status. And child under-nutrition among employed mothers was affected by feeding frequency, a child’s immunization status, and having been sick during the previous two weeks.

This study found that the prevalence of under nutrition was significantly higher among male sex children than female sex children, which is consistent with a study conducted in Sri Lanka on the nutritional status of children aged 6–24 months [26] and other studies conducted in Kenya, Nigeria, and Myanmar where sex was identified as a significant predictor of nutritional status, with boys having worse nutritional status than girls [27–29]. This differences in nutritional status between male and female children might be due to the fact that mothers provide more protection and attention for girls than boys thus they were much less likely leave girls with an alternate caregiver than boys [30]. So that Girls were much more likely to be cared for with inadequate child care than were boys which more likely improves nutritional status of girls than boys. It could be also due to biological differences in morbidity between boys and girls in their early life. Males have been found to have a higher risk of morbidity throughout perinatal, infancy, and childhood [31], which will make male children more likely to suffer from under nutrition. It is also observed that children’s age was associated with under-nutrition. As children age increase the odds of being under-nourished decreases significantly. This is in line with a study conducted in South and Northern Ethiopia [21, 22] and opposed to the result of a study in Pakistan [32] the reason behind might be young children may have bacterial infections resulting in diarrhea which could in turn cause high nutrient loss from their body. On the other hand, older age children will usually consume varieties of foodstuffs thereby preventing undernutrition.

In the current study, under-nutrition among children of unemployed women was found significantly associated with household food insecurity. Children from food insecure household were more likely to be under nourished than children from food secured household. This finding is in line with some of the studies conducted in India, South Africa and South Ethiopia [22, 33–35]. This might be due to children from food insecure household could not access enough food intake and reduced dietary diversity, that may results in malnutrition in general. Furthermore, the odds of under-nutrition among children of employed women who had low meal frequency for their age were high. This result was supported by studies done in Afghanistan [36] and southern Ethiopia [37], which reported significantly higher proportion of under-nutrition among children who had low meal frequency per day. The possibly justification might be due attention were not be given to children in the household in terms of age-appropriate feeding, and adequate meal frequency by parents.

Antenatal care visit of mother during pregnancy is one of the factors that affect under-nutrition among children of unemployed mothers. The result shows the fact that children born to mothers who don’t attend antenatal care visit during their pregnancy were more likely to be under nourished compared to their counterparts which is supported by a study in Bangladesh which assessment of the association between antenatal care and child malnutrition [38]. This might be the advice they obtain during antenatal visit helps them to take care of their children. Although the vaccination and medicine they take from the service delivery helps the children resistant to disease. On the side of unemployed mothers, exclusive breast feeding up to age of six month also shows a significant effect on the child’s nutritional status.

In this study, children of employed women who failed to receive all vaccinations for their age, as recommended by the national immunization program, showed positive association with under-nutrition. This result is consistent with previous studies from Thailand [39], South Asian countries [40], Ethiopia [41] and pooled result of 16 countries DHS report [42] reporting that children who were partially vaccinated were more likely to be undernourished than fully vaccinated children. The result suggests that immunization not only helps to prevent specific disease of focus but also have a protective effect against malnutrition via increasing the resistant to disease and leads to overall improvements in health. In relation to this, this study also revealed that children of employed women who had been sick during the previous two weeks were more likely to have under-nutrition. This finding is consistent with other studies [43–45]. This may be due to the fact that becoming ill accelerates the onset of malnutrition by reducing food intake and its utilization via increasing nutrient loss and catabolic reactions in the organism. Though this study came up with this finding after a thorough investigation following scientific procedures, it will not be free from limitations, and its findings should be interpreted cautiously. Social desirability bias and recall bias in 24-hour recall dietary assessment might affect the outcomes of this study to some extent.

### Conclusion and recommendation

In conclusion, the prevalence of under-nutrition among children of unemployed mothers is significantly higher than those children of employed mothers which consolidate the evidence that mother’s employment status have a positive association with child nutrition. Overall, maternal employment, child sex, child age by one month, ANC follow up during pregnancy, child got sick in the last two weeks prior data collection, food security status, feeding frequency, and food diversity were found to be significant predictors of child under-nutrition. However, in a separate model, different factors were identified as significant predictors of child under-nutrition among these two groups (employed and unemployed women). While nutritional status of children of unemployed mothers is affected by sex of the child, age of the child, food security status of the household, ANC follow-up during pregnancy, and six month exclusive breast feeding status. And nutritional status children of employed mothers affected by sex of the child, age of the child, feeding frequency, child’s immunization status, and having been sick during the previous two weeks. Thus, the government focus of women empowerment should have to be continued via employing mothers and multi-sectoral approach intervention together with agriculture and education offices should be strengthened. Tailored interventions addressing modifiable risk factors of child under-nutrition such as ANC follow-up during pregnancy, child immunization, and exclusive breastfeeding should also be devised so as to improve nutritional status of children in the study area. Scholars are recommended to conduct a study using strong designs like prospective study design to explore factors that are not addressed in this study like maternal nutrition during pregnancy.

## Data Availability

All the required data will be available upon reasonable request to the corresponding author.

## Abbreviations

ANC: Antenatal Care
DHS: Demographic and Health Survey
EDHS: Ethiopian Demographic and Health Survey
HFIA: Household Food Insecurity Access
LMICs: Low and Middle Income Countries
MUAC: Mid Upper Arm Circumference
NGO: Non-governmental Organization
NNP: National Nutrition Program
PCA: Principal Component Analysis
WHO: World Health Organization
WLZ: Weight-for-Length Z score
